# Development and Validation of a Stable Isotope Dilution Headspace–SPME–GC/MS Method for the Determination of Vanillin in Fragrant Vegetable Oils

**DOI:** 10.3390/molecules28217288

**Published:** 2023-10-26

**Authors:** Fangyi Mei, Hongling Wang, Yuquan Zhang, Mei Zhang, Shuai Zhou, Haiming Shi, Yuanrong Jiang

**Affiliations:** 1 Wilmar (Shanghai) Biotechnology Research & Development Center Co., Ltd., Shanghai 200137, Chinazhoushuai860311@163.com (S.Z.); 2 Yihai Kerry (Qingdao) Oils & Grains Industries Co., Ltd., Qingdao 266321, China

**Keywords:** vanillin, fragrant vegetable oil, sesame oil, peanut oil, rapeseed oil, stable isotope dilution assay, HS–SPME–GC/MS

## Abstract

It has been reported that vanillin has been intentionally added to enhance the taste and flavor of low-quality vegetable oils. Therefore, it is crucial to investigate the accurate concentrations of vanillin in three types of fragrant vegetable oils commonly consumed in China. In this study, a method has been developed for the quantification of vanillin in commercial fragrant vegetable oils using the stable isotope dilution assay (SIDA) and headspace–solid-phase microextraction (HS–SPME) coupled with gas chromatography/mass spectrometry (GC/MS). The limit of detection (LOD) and limit of quantification (LOQ) of the analyte were determined to be 20 µg kg^−1^ and 50 µg kg^−1^, respectively. The validation study demonstrated that the recoveries ranged from 89% to 101%, with intra-day and inter-day precision being less than 7.46%. A survey of 80 commercially available fragrant vegetable oils was performed using the present method. Vanillin was found to be widely present in fragrant vegetable oils, with sesame oils showing the highest average content (842.6 µg kg^−1^), followed by rapeseed oils (262.1 µg kg^−1^) and peanut oils (115.0 µg kg^−1^). The results indicate that the proposed method is a simple, accurate, and eco-friendly approach for determining the presences of vanillin in fragrant vegetable oils.

## 1. Introduction

Vanillin, chemically known as 4-hydroxy-3-methoxybenzaldehyde, is an important flavor compound that is renowned for its pleasant sweet and unique natural milk flavor [1,2]. It is commonly employed as a flavor enhancer or stabilizer to enhance the aroma of various foods items, such as candies, cakes, chocolates, milk powder, beverages, and wines [3,4,5,6,7]. Numerous studies have demonstrated the broad spectrum of bioactivities exhibited by vanillin, including anticancer, antidiabetic, antioxidant, anti-sickling, antimicrobial, anti-inflammatory, aphrodisiac, cardioprotective, and diuretic properties [8,9]. However, existing studies have also highlighted the potential adverse effects of excessive vanillin consumption, such as headache, nausea, vomiting, breathing difficulties, and even damage to the liver and kidneys [2,10,11]. In recent years, several studies have reported the illegal addition of vanillin to enhance the taste and flavor of low-quality vegetable oils or other inexpensive substitutes [12,13,14]. Consequently, the Chinese regulatory authorities have implemented stringent food safety regulations (Chinese National Food Safety Standard, GB 2760-2014) [15] to prohibit the inclusion of artificial flavors, including vanillin and its derivatives, in vegetable oils. However, vanillin was discovered as an endogenous compound in sesame and can be transferred to sesame oil during processing, making it an important marker for the quality control of industrial processes [14]. Furthermore, vanillin has also been found in other kinds of vegetable oils, such as virgin olive oil, flaxseed oil, tea seed oil, rapeseed oil, and rice bran oil [14,16,17,18,19]. These findings have raised doubts regarding the reliability of using vanillin as a marker for the detection of adulteration in fragrant vegetable oils, since they originally contained vanillin. Therefore, it is imperative to develop a simple and reliable method for detecting vanillin in vegetable oils.

Existing analytical methods developed thus far for quantifying vanillin in vegetable oils include high-performance liquid chromatography (HPLC) with diode array detection (DAD) [20,21], high-performance liquid chromatography–tandem mass spectrometry (HPLC–MS/MS) [12,22], ultra-performance liquid chromatography–tandem mass spectrometry (UPLC–MS/MS) [13,14,23], and high-performance liquid chromatography coupled with time-of-flight mass spectrometry (HPLC–ESI-TOF-MS) [17,24]. Most of these methods are time consuming and face analytical challenges, requiring a large quantity of organic solvents. Furthermore, the determination of trace amounts of vanillin in vegetable oils remains challenging due to the matrix effect of triglycerides [25]. To address some of these issues, a method utilizing headspace–solid-phase microextraction coupled with gas chromatography/mass spectrometry (HS–SPME–GC/MS) has recently been developed to identify vanillin in fragrant vegetable oil [11]. However, in a previous study, this method employed a manual SPME holder without precise temperature control and orbital agitation during incubation, leading to potential fluctuation in the analytical results [26,27,28]. Moreover, the SPME optimization process in the recent study did not consider the matrix effect of the real sample, and the methodology validation was not comprehensive [11]. A more robust analytic solution is desirable for determining vanillin in fragrant vegetable oils.

The objective of this study was to develop a simple, accurate, and high-throughput method for quantifying vanillin in fragrant vegetable oils using HS–SPME–GC/MS. To achieve this, the Plackett–Burman design and central composite rotational design (CCD) were employed to optimize the extraction conditions of the target compound. We validated our approach using GC–MS with a stable isotope internal standard in two commercially available samples with different levels of vanillin. The application of our method was tested on three types of fragrant vegetable oils (including 40 sesame oil (SSO) samples, 20 peanut oil (PNO) samples, and 20 rapeseed oil (RSO) samples) with resulting differences among the three types, which was consistent with previous reports [12,22]. Some of the results were compared with those obtained using the aforementioned UPLC–MS/MS methods. To the best of our knowledge, this is the first report on the application of automatic HS–SPME–GC/MS with a selected ion monitoring mode for the accurate quantitative analysis of vanillin in fragrant vegetable oils.

## 2. Results and Discussion

### 2.1. Optimization of the HS–SPME–GC/MS Method

#### 2.1.1. Screening of Significant Factors (Plackett–Burman Design)

**Figure 1:**
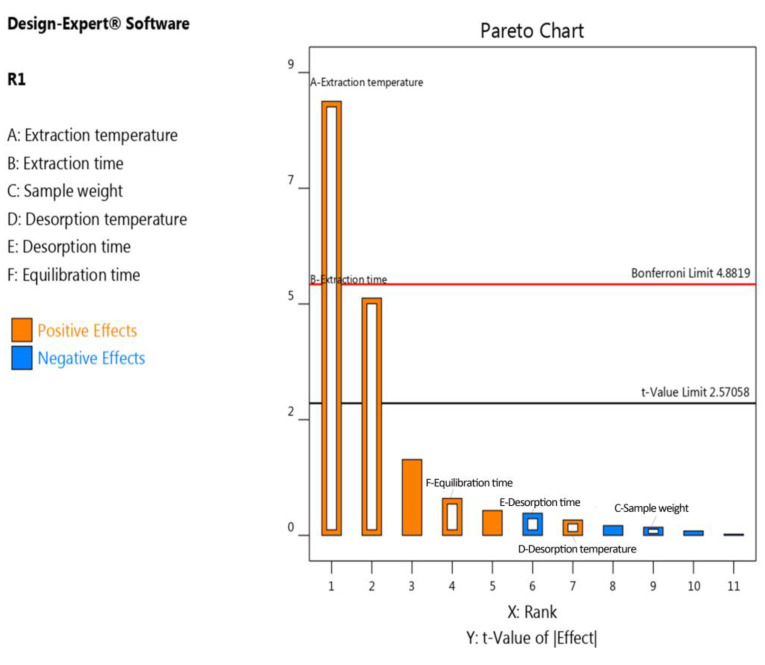
Pareto diagram for the total areas of the vanillin extracted from sesame oil.

The results obtained from the Plackett–Burman (PB) design are summarized in the Pareto chart (Figure 1, Table S2), indicating that the extraction time and extraction temperature were the most significant variables. Other factors such as sample weight, equilibration time, desorption temperature, and desorption time did not show a significant effect, suggesting that they can be fixed at any value, since adjusting them would have an equal effect on the extraction of vanillin. Therefore, only extraction time and extraction temperature were further evaluated in the optimization process using the response surface methodology (CCD).

#### 2.1.2. Single-Factor Experiment

Different chemical compounds require specific types of SPME fibers as each fiber possesses unique absorption/adsorption properties, making the choice of fiber crucial for optimal extraction efficiency. To determine the optimal extraction effect for vanillin extraction from fragrant vegetable oils using HS–SPME–GC/MS, four commercial SPME fibers (50/30 μm DVB/CAR on PDMS, 65 μm PDMS/DVB, 75 μm CAR/PDMS, and 100 μm PDMS) were tested, respectively. The overall extraction effect of the selected SPME fiber was evaluated based on the peak area of vanillin under the following conditions: 4 g sample weight, 15 min equilibration time, 110 °C extraction temperature, 45 min extraction time, 280 °C desorption temperature, and 1.5 min desorption time.

**Figure 2:**
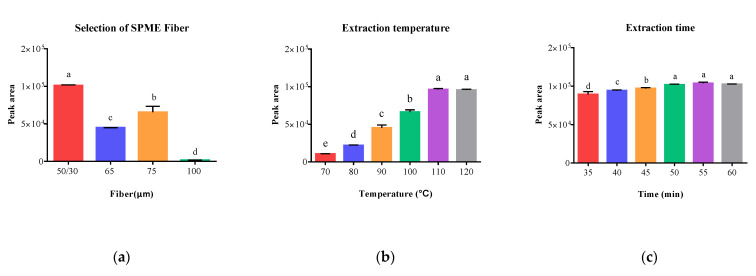
Effects of different parameters on the extraction efficiency of vanillin in sesame oil, including extraction fiber (**a**), extraction temperature (**b**), and extraction time (**c**). Note: a–e different superscripted letter in the same column means significant differences (*p* < 0.05).

As is shown in Figure 2a, 50/30 μm DVB/CAR on PDMS fiber exhibited the highest peak area of 1.01 × 10^5^. These results indicated that mixed-phase coatings (50/30 μm DVB/CAR on PDMS, 65 μm PDMS/DVB, and 75 μm CAR/PDMS) produced significantly higher peak areas compared to single-phase fibers (100 μm PDMS), suggesting that mixed-phase coatings offer complementary properties compared to single-phase films, enabling them to adsorb a wide range of analytes with diverse chemical properties.

According to the theory of SPME, increasing the extraction temperature can enhance the extraction efficiency. However, excessively high extraction temperatures can decrease the distribution coefficient of analytes between the fiber and the sample, potentially leading to matrix reactions [28]. Six different temperatures (70, 80, 90, 100, 110, and 120 °C) were investigated to determine the optimal extraction temperature, conducted using a 50/30 μm DVB/CAR on PDMS fiber, 4 g sample weight, 15 min equilibration time, 45 min extraction time, 280 °C desorption temperature, and 1.5 min desorption time.

The results illustrated in Figure 2b demonstrated a significant increase in peak areas with increasing temperature from 70 to 110 °C. However, there was no substantial increase in peak areas when the temperature was further increased to 120 °C, and higher temperatures can also reduce the fiber’s lifespan. Consequently, 110 °C was considered to be the appropriate temperature for subsequent experiments.

In the SPME process, the concentrations of analytes in the sample matrix and extractant reach an equilibrium. The amount of vanillin adsorbed onto the fiber increases with an increase in extraction time until an equilibrium is reached [29]. Hence, a series of extraction times ranging from 35 to 60 min with 5 min increments were investigated. Other conditions remained fixed: 50/30 μm DVB/CAR on PDMS fiber, 4 g sample weight, 15 min equilibration time, 110 °C extraction temperature, 280 °C desorption temperature, and 1.5 min desorption time. As expected, the peak areas of vanillin increased with longer extraction times and eventually reached a stable level (Figure 2c). When the extraction time was set at 55 min, the peak areas approached the maximum and remained stable, with a slight fluctuation when the extraction time was extended to 50 min. Furthermore, there was no significant difference between an extraction time of 50 min, 55 min and 60 min. Therefore, an extraction time of 50 min was chosen for subsequent experiments.

#### 2.1.3. Optimization of Significant Factors

In the initial stage of the optimization process, the PB design identified extraction temperature and extraction time as the significant factors requiring further optimization using a central composite design in RSM. The variables and their low, middle, and high levels were as follows: extraction temperature (100, 110, 120 °C) and extraction time (45, 50, 55 min). Thirteen experiments were conducted, and the results are summarized in Table S3. As is shown in Figures S1 and S2, the optimal extraction temperature and time were determined to be 109.47 °C and 55 min, respectively, resulting in a vanillin peak area of 9.95 × 10^4^. For ease of operation, the extraction parameters were adjusted to 110 °C for 55 min, yielding an actual peak area of 1.03 × 10^5^. A comparison between the simulated and actual values demonstrated a close agreement (CV = 3.71%).

Taken together, the optimized extraction conditions for achieving the best response were as follows: 50/30 μm DVB/CAR on PDMS fiber, 110 °C extraction temperature, and 55 min extraction time. Other working extraction conditions included a 4 g sample weight, 15 min equilibration time, 280 °C desorption temperature, and 1.5 min desorption time.

### 2.2. Method Validation

For laboratory validation, the optimized method was studied following European Commission Decision 2002/657/EC and JRC Technical Reports with minor modifications [30,31]. The analytic parameters evaluated included linearity, limit of detection (LOD), limit of quantification (LOQ), accuracy, and intra-day and inter-day precision. To demonstrate the applicability of the method, recovery experiments were performed by spiking two types of fragrant sesame oils with vanillin standard solutions.

#### 2.2.1. Calibration, LOD, and LOQ

As there is no readily available vanillin-free lipid matrix, caprylic capric triglyceride (ODO) was used to prepare the calibration curve. For the analysis of oil samples, a stock solution of a mixed standard (1.00 × 10^6^ μg kg^−1^) containing vanillin was prepared by dissolving 10.0 mg of vanillin in 10 g of ODO, and the resulting mixture was stored at 4 °C. The stable isotope labeled as the internal standard, [^13^C_6_]-vanillin, was also prepared using the same procedure. The calibration curves were constructed by plotting the peak area ratios of the target compound relative to the corresponding internal standard against the gradient concentrations. The standards for the calibration curves were prepared by progressive dilution of the stock solution of the standard, while the internal standards remained at a fixed concentration (500 μg kg^−1^).

Under the optimized conditions, the vanillin standard solutions were analyzed at the concentrations ranging from 50 to 5000 μg kg^−1^. The calibration curves offered satisfactory linearity (r^2^ ≥ 0.9999). The limits of detection (LOD) and quantification (LOQ) were determined based on a signal-to-noise ratio (S/N) of approximately 3 and 10, respectively. The LOD values for vanillin were 20.0 μg kg^−1^, and the LOQ values were 50.0 μg kg^−1^.

#### 2.2.2. Precision and Recovery

The established method demonstrated good reproducibility for the quantification of vanillin, with intra- and inter-day variations of less than 6.4% and 7.5%, respectively (Table 1). The accuracy of the method was confirmed through recovery experiments. Three different quantities (50%, 100%, and 200%) of vanillin were added to a certain amount of sesame oil. The resulting samples were extracted and analyzed using the methods described above. The quantity of vanillin was determined directly using the calibration equation. As is shown in Table 1, the recovery values, estimated from the measured versus added amounts, ranged from 89.1 to 92.8% and from 95.1 to 101.9% for vanillin in the two respective real samples, indicating good accuracy. When comparing the present method to the HS–SPME–GC/MS method used in previous studies, Peng et al. (2019) reported vanillin recoveries in sesame oil ranging from 86.0% to 88.0% using an SPME-GC/MS method with a manual holder, and their study did not include a validation of reproducibility [11]. Furthermore, their SPME optimization was performed using a standard solution. In contrast, the proposed method in this study exhibited a simpler pretreatment procedure, increased automatic control of temperature and vibration, better recoveries, and a more comprehensive validation of the methodology in the complex lipid matrix.

#### 2.2.3. Comparison of Vanillin Content in SSO Determined by Two Methods

To further validate the established method, the content of vanillin in fragrant vegetable oils was determined using both the stable isotope dilution HS–SPME–GC/MS and LC–MS/MS methods. To assess the statistical significance of any differences between the two methods, the percentage difference (Equation (1)) between the results obtained from the two quantification methods was calculated as a measure of the method’s accuracy for vanillin quantification. The results in Table 2 demonstrate good agreement between the two quantification methods, indicating that there was no significant difference between the results obtained from the two methods [32].

(1) $$Difference(\%)=\frac{\left|measured_{1}-measured\left.{}_{2}\right|\right.}{\left(\frac{measured_{1}+measured_{2}}{2}\right)}\times 100$$

### 2.3. Quantification of Vanillin in Fragrant Vegetable Oils

**Figure 3:**
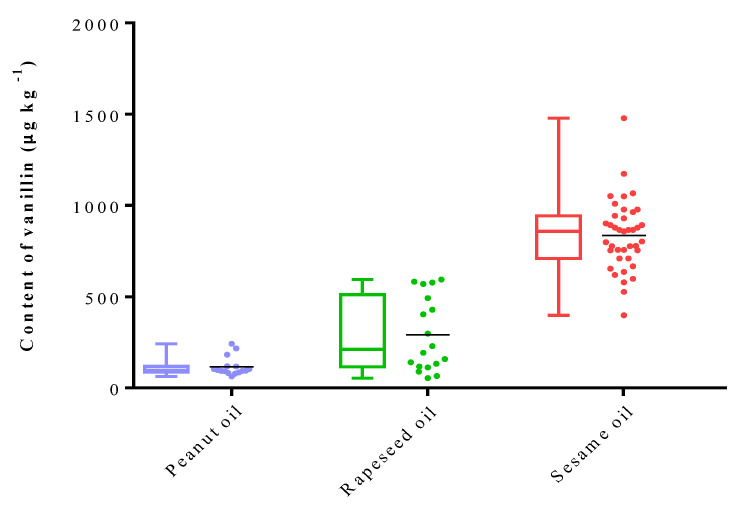
Contents (μg kg^−1^) of vanillin in three different types of flavored oils (peanut oil (PNO), rapeseed oil (RSO) and sesame oil (SSO)).

In total, 80 commercial fragrant vegetable oils, comprising 40 samples of SSO, 20 samples of PNO, and 20 samples of RSO, were analyzed using the established HS–SPME–GC/MS method in this study. SSO exhibited the highest average concentration of vanillin at 842.6 μg kg^−1^, while RSO displayed a moderate average vanillin concentration of 262.1 μg kg^−1^. This was followed by PNO with a lower vanillin concentration of 115.0 μg kg^−1^ (Figure 3). This observation is consistent with a previous report by Qu et al. [22]. Among these samples, SSO showed significant variation in the vanillin content, ranging from 398.0 to 1479.4 μg kg^−1^. These variations may be attributed to differences in sesame genotypes, planting environments, processing techniques, and storage conditions.

## 3. Materials and Methods

### 3.1. Materials and Reagents

Vanillin standard (99.9%) was purchased from Sigma-Aldrich (St. Louis, MO, USA). [^13^C_6_]-Vanillin (99%) was obtained from Cambridge Isotope Laboratories (Andover, MA, USA). HPLC-grade acetonitrile, methanol, and formic acid were purchased from Fisher Scientific (Fair Lawn, NJ, USA). Ultrapure water was prepared using a Milli-Q system obtained from Millipore (Billerica, MA, USA). Caprylic capric triglyceride (ODO), utilized for preparing the calibration curves, was purchased from Titan Chemical Co., Ltd. (Shanghai, China). Other chemicals were procured from Anpel Laboratory Technologies (Shanghai, China). All SPME fibers, along with clear-glass screw cap vials (PTFE/silica septa) were acquired from Supelco (Bellefonte, PA, USA).

### 3.2. Preparation of Oil Samples

To investigate the distribution of vanillin content in various types of fragrant vegetable oils, a total of 80 samples were procured from various local supermarkets and online retail stores. The comprehensive details regarding these samples (forty sesame oil samples, twenty peanut oil samples, and twenty rapeseed oil samples) can be found in the Supplementary Materials (Table S1). Each oil sample was assigned a distinct number and stored in darkness at −20 °C until further analysis.

### 3.3. Optimization of the HS–SPME–GC/MS Method

#### 3.3.1. SPME Optimization (Experimental Design Approach)

The optimization of the SPME method was conducted in three stages: screening, single-factor experiments, and optimization. The screening stage utilized the Plackett–Burman design, followed by the single-factor experiment. The optimization stage employed the response surface methodology (RSM) with a central composite design. The experimental designs for both the screening and optimization stages were generated using Design-Expert version 6.0.4 (Stat Ease Software, Minneapolis, MN, USA).

- Screening of significant factors (Plackett–Burman design)

The HS–SPME conditions were optimized using the Plackett–Burman (PB) design, which served as a methodology for identifying the variables that could impact the extraction of vanillin from fragrant vegetable oils. Subsequently, a central composite rotational design (CCD) was employed, utilizing a 2^6^ factorial design with 13 axial points and 3 repetitions of the central point, to determine the optimal extraction conditions. The selected variables for the PB design included sample weight (g), equilibration time (min), desorption temperature (°C), extraction time (min), extraction temperature (°C), and desorption time (min). Table 3 displays the factor levels and the experimental domain of the PB design.

The Plackett–Burman design matrix consisted of the following factors: extraction temperature (80 °C and 120 °C), extraction time (10 min and 50 min), sample weight (3 g and 7 g), desorption temperature (240 °C and 260 °C), desorption time (3 min and 7 min), and equilibration time (5 min and 25 min). The significant factors identified through the Plackett–Burman design were subsequently subjected to further optimization using the response surface methodology (RSM).

- Single-factor experiment

Several parameters, such as the SPME fiber coating and extraction conditions, can significantly impact the extraction efficiency, detection sensitivity, selectivity, and reproducibility. Hence, these parameters were meticulously optimized, and the resulting data were subjected to analysis through a one-way analysis of variance (ANOVA).

- Optimization of significant factors

The significant factors identified during the screening stage, namely extraction temperature and extraction time, were subjected to further optimization using the response surface methodology (RSM) with a central composite design (CCD). Considering their frequent occurrence and relevance in early preliminary studies, the peak area of vanillin was selected as the response variable for the CCD design.

#### 3.3.2. HS–SPME–GC/MS Analysis

The HS–SPME system is widely employed for extracting flavor compounds. In this study, automatic HS–SPME experiments were conducted using a Divinylbenzene–Carboxen–Polydimethylsiloxane fiber (50/30 µm DVB/CAR/PDMS) (Supelco, Bellefonte, PA, USA) on a CTC Combi PAL TM autosampler (Zwingen, Switzerland). The autosampler was equipped with an SPME fiber conditioning station, a sample tray capable of holding 32 vials of 20 mL, and a temperature-controlled single magnet mixer tray (SMM tray) (Chromtech, Idstein, Germany). Prior to the experiments, extraction conditions were optimized independently. Four grams of sesame oil were weighed into a 20 mL vial, which was tightly capped with a PTFE/silicone septum. After 5 min of equilibration at 60 °C, the SPME fiber was exposed to the vial headspace for 20 min under orbital shaking at 500 rpm. Subsequently, the fiber was immediately desorbed for 2 min in a GC injection port operating in the splitless mode at 250 °C. The fiber was conditioned at 300 °C under helium flow for 1 h in accordance with the manufacturer’s recommendations prior to use. Periodic fiber blanks were run to ensure the absence of contaminants and carryover.

An Agilent 7890A GC coupled with an Agilent 5975 MSD was utilized for separating and identifying the volatile compounds of fragrant vegetable oils. The GC system was equipped with a 30 m × 0.25 mm id HP-5-MS capillary column with a 0.25 μm film thickness (J&W Scientific, Folsom, CA, USA). Helium was used as the carrier gas at a flow rate of 1 mL min^−1^. The column temperature program was as follows: temperature was held at 110 °C for 0.5 min, followed by a constant rise to 130 °C at 5 °C min^−1^; then, it was increased to 170 °C at 2 °C min^−1^ and held for 1 min, further increased to 180 °C at 10 °C min^−1^, and finally, raised to 280 °C at 30 °C min^−1^, with a post-run at 290 °C for 1 min.

To improve the detection effectiveness, three different chromatographic columns with varying polarity were employed: HP-5, DB-17-ms, and DB-WAX (J&W Scientific, Folsom, CA, USA; 30 m × 0.25 mm × 0.25 μm film thickness). The results revealed that HP-5 exhibited a superior separation efficiency for the volatile compounds. The GC–MS interface and ion source temperatures were set at 280 °C and 230 °C, respectively. Electron impact mass spectra were generated at 70 eV. The mass spectrometer was operated in the selected ion monitoring (SIM) mode after identifying the relevant peaks in the full scan mode, and the mass spectra for the scan were recorded at 70 eV from *m*/*z* values of 40 to 400 amu. The analytes were quantified using isotopically labeled internal standards. For quantification and confirmation, ions with *m*/*z* values of 150.9, 108.9, and 122.8 were selected to monitor vanillin, while *m*/*z* values of 157.0, 115.0, and 158.0 were chosen for [^13^C_6_]-vanillin. Agilent ChemStation software (version E.02.02.1431) was utilized for data acquisition. The aforementioned instrumentation and chromatographic conditions were employed to develop and validate the vanillin analysis. Each sample was analyzed in triplicate using HS–SPME–GC/MS.

### 3.4. Method Validation

#### 3.4.1. Calibration, Limit of Detection (LOD) and Limit of Quantification (LOQ)

Stock solutions of vanillin were diluted to appropriate concentrations within the range of 50–5000 µg kg^−1^ to establish the calibration curves. The testing standard solutions were analyzed in triplicate. The limits of detection (LOD) and quantification (LOQ) were measured as the signal-to-noise ratios (S/N) of 3 and 10, respectively. Calibration curves were constructed by plotting the peak areas against the analyte concentrations. The calibration curve for vanillin was represented by the equation y = 987.8x + 83.6.

#### 3.4.2. Precision and Recovery

To validate the method’s applicability, two types of sesame oil with high (892.0 μg kg^−1^) and medium (610.4 μg kg^−1^) levels of vanillin were investigated. Precision was measured by intra- and inter-day variabilities. The vanillin standard solutions with low, medium, and high concentrations were analyzed to calculate the relative standard deviation (RSD). The quantitation of vanillin was determined using the external standard method. Intra-day variability was evaluated by analyzing the standard solution in six replicates within a single day. Inter-day variability was assessed by analyzing the standard solution in six replicates over three consecutive days.

#### 3.4.3. UPLC–MS/MS Analysis

The China Food and Drug Administration standards (BJS201705, Determination of vanillin, methyl vanillin, ethyl vanillin, and coumarin in foods) were applied in this study with minor modifications. An Agilent (Palo Alto, CA, USA) 6460 series triple-quadrupole UPLC–MS/MS with an electrospray ionization (ESI) interface was used in the positive ionization mode. Data were collected using Masshunter software version A.09.03. The separation was performed on an Agilent Zorbax Eclipse Plus C18, Rapid Resolution HD column (150 × 2.1 mm, with a particle size of 1.8 μm). The column temperature was maintained at 30 °C. The mobile phase consisted of water (0.1% formic acid) (A) and MeOH (B). The gradient elution program was as follows: 0–0.5 min, 70% A; 0.5–2.0 min, 75% A; 2.0–5.0 min, 65% A; and 5.1–8.0 min, 5% A. The injection volume was 4 μL, and the flow rate was 0.30 mL min^−1^. MS analyses were performed using multiple reaction monitoring (MRM) with an ESI source operated in the positive ionization mode. The operating conditions were as follows: ion spray voltage 4.0 kV; ion source temperature 300 °C; curtain gas 40 psi; and auxiliary gas 40 psi. Quantitative ion pairs for vanillin and vanillin-^13^C were observed at *m*/*z* 153.1→*m*/*z* 93.1 and *m*/*z* 159.0→*m*/*z* 99.0, respectively.

### 3.5. Statistical Analysis

Data were reported as the mean ± SD for triplicate determinations. Graphs were created on Graph Pad Prism (Version 6.07, Graph Pad Software Inc., San Diego, CA, USA). A Student’s *t*-test was performed with a *p*-value of 0.05. The results were employed to detect differences between the two methods. Statistical analysis was performed using SPSS for Windows (version 17.0.0, 2008, SPSS Inc., Chicago, IL, USA).

## 4. Conclusions

Vanillin was detected with a wide concentration range in three types of fragrant vegetable oils using HS–SPME–GC/MS with a stable isotope internal standard. The results of this study provide valuable insights and serve as a comprehensive reference for the state or local administration to develop appropriate regulations and prevent the illegal addition of vanillin in fragrant vegetable oils available on the Chinese market. Our proposed method also benefits law enforcement agencies and vegetable oil manufacturers by enabling them to monitor concentration changes in vanillin during processing and by promoting the effective quality control of their products.

## Figures and Tables

**Table 1:** Precision and recovery for the proposed HS–SPME–GC/MS method.

Samples	Concentration (μg kg^−1^)	Intra-Day Precision (%RSD)	Inter-Day Precision (%RSD)	Recovery (%)
SSO-25	950.1	6.40	2.07	95.13
	1190.0	2.09	6.59	97.48
	1860.4	4.36	6.09	101.90
SSO-31	1350.3	2.34	7.46	89.14
	1850.1	3.81	2.30	90.23
	2760.2	1.55	1.62	92.79

**Table 2:** Comparison of the vanillin content (μg kg^−1^) in fragrant vegetable oils determined by LC–MS/MS and HS–SPME–GC/MS *.

Samples	Vanillin (μg kg^−1^)		
	LC–MS/MS	HS–SPME–GC/MS	Difference (%)
Sesame oil 1	947.0a ± 15.5	963.7a ± 8.0	1.80
Sesame oil 9	641.3a ± 11.1	636.3a ± 14.6	0.85
Sesame oil 12	865.6a ± 20.0	858.3a ± 16.5	0.88
Sesame oil 27	1082.0a ± 55.2	1113.3a ± 55.1	2.82
Sesame oil 28	886.0a ± 16.0	879.0a ± 16.0	0.79
Rapeseed oil 4	212.3a ± 12.7	229.0a ± 2.7	7.74
Rapeseed oil 8	550.6a ± 15.1	577.0a ± 7.0	4.68
Rapeseed oil 10	394.3a ± 13.0	403.2a ± 11.6	2.26
Rapeseed oil 12	105.2a ± 2.6	113.4a ± 5.8	7.50
Rapeseed oil 17	131.3a ± 2.1	140.0a ± 7.0	6.48
Peanut oil 3	109.4a ± 8.4	118.3a ± 2.9	8.41
Peanut oil 6	95.9a ± 2.3	99.3a ± 4.5	3.79
Peanut oil 10	110.5a ± 2.9	118.7a ± 2.3	7.58
Peanut oil 15	262.0a ± 5.0	242.3a ± 2.1	7.75
Peanut oil 19	89.4a ± 3.7	93.0a ± 6.1	4.05

* Values represent the mean of the triplicate analyzes ± the standard deviation. a—Values in the same row marked by different letters are significantly different (*p* < 0.05).

**Table 3:** Factor levels and experimental domain applied to the Placket–Burman experimental design.

Factor	Experimental Domain		
	−1	0	1
Extraction temperature (°C)	80	100	120
Extraction time (min)	10	30	50
Sample weight (g)	3	5	7
Desorption temperature (°C)	240	250	260
Desorption time (min)	3	5	7
Equilibration time (min)	5	15	25

## Data Availability

Not applicable.

## Supplementary Materials

The following supporting information can be downloaded at: https://www.mdpi.com/article/10.3390/molecules28217288/s1, Figure S1. Three-dimensional surface plot from Plackett–Burman design; Figure S2. The ramps from Plackett–Burman design; Table S1. Detailed information of fragrant vegetable oils used in this study; Table S2. PB experimental design and summary of results; Table S3. CCD experimental design and summary of results.
