# Supplementary material for: Development and Validation of a Stable Isotope Dilution Headspace–SPME–GC/MS Method for the Determination of Vanillin in Fragrant Vegetable Oils
Source: Molecules. 2023 Oct 26;28(21):7288. doi: 10.3390/molecules28217288 (PMC10650462; doi:10.3390/molecules28217288)
Supplement: Supplementary file 1 [file molecules-28-07288-s001.zip › molecules-2669279-supplementary.pdf]

Supplementary Information

Contents:

Figure S1. 3D surface plot from Plackett-Burman design

Figure S2. The ramps from Plackett-Burman design

Table S1. Detailed information of fragrant vegetable oils used in this study

Table S2. PB experimental design and summary of results

Table S3. CCD experimental design and summary of results

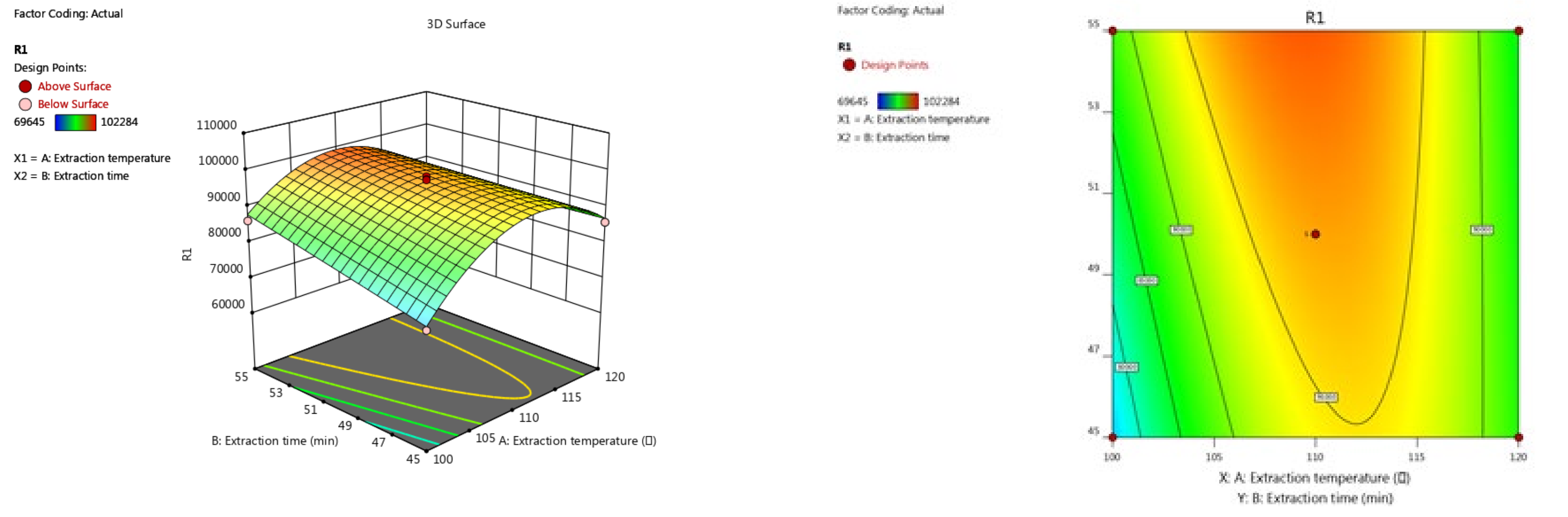

Figure S1. 3D surface plot from Plackett-Burman design.

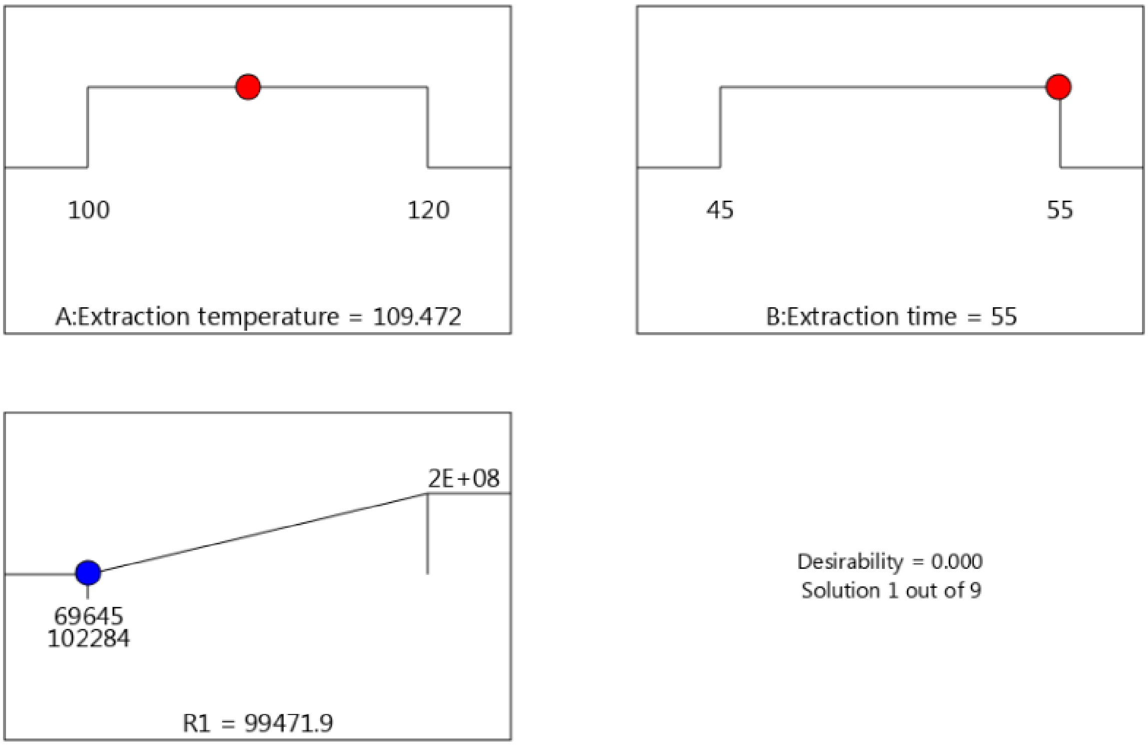

Figure S2. The ramps from Plackett-Burman design.

Table S1. Detailed information of fragrant vegetable oils used in this study.

Vegetable Oils	Producer origin	Technology	Vanillin (μg/kg)
Sesame oil 1	Shandong, China	Hot Pressed	963.7
Sesame oil 2	Shandong, China	Hot Pressed	978.0
Sesame oil 3	Shandong, China	Hot Pressed	776.8
Sesame oil 4	Hubei, China	Hot Pressed	865.7
Sesame oil 5	Shandong, China	Hot Pressed	930.1
Sesame oil 6	Henan,China	Hot Pressed	1066.9
Sesame oil 7	Anhui,China	Hot Pressed	757.2
Sesame oil 8	Anhui,China	Hot Pressed	710.3
Sesame oil 9	Anhui,China	Hot Pressed	636.3
Sesame oil 10	Hunan, China	Hot Pressed	1479.4
Sesame oil 11	Shandong, China	Hot Pressed	1173.7
Sesame oil 12	Shandong, China	Hot Pressed	858.3
Sesame oil 13	Beijing, China	Hot Pressed	526.7
Sesame oil 14	Shandong, China	Hot Pressed	943.2
Sesame oil 15	Shandong, China	Hot Pressed	900.8
Sesame oil 16	Shandong, China	Hot Pressed	757.3
Sesame oil 17	Shandong, China	Hot Pressed	710.0
Sesame oil 18	Shandong, China	Hot Pressed	978.0
Sesame oil 19	Shandong, China	Hot Pressed	778.3
Sesame oil 20	Shandong, China	Hot Pressed	866.4
Sesame oil 21	Hebei, China	Hot Pressed	777.2
Sesame oil 22	Hebei, China	Hot Pressed	866.5
Sesame oil 23	Anhui, China	Hot Pressed	398.0
Sesame oil 24	Tianjin, China	Hot Pressed	666.3
Sesame oil 25	Tianjin, China	Hot Pressed	610.4
Sesame oil 26	Shandong, China	Hot Pressed	654.0
Sesame oil 27	Anhui, China	Hot Pressed	1113.3
Sesame oil 28	Shandong, China	Aqueous extraction	879.0
Sesame oil 29	Shandong, China	Aqueous extraction	798.2
Sesame oil 30	Shandong, China	Aqueous extraction	754.3
Sesame oil 31	Shandong, China	Aqueous extraction	892.0
Sesame oil 32	Shandong, China	Aqueous extraction	1010.0
Sesame oil 33	Shandong, China	Aqueous extraction	1050.2
Sesame oil 34	Shandong, China	Aqueous extraction	879.0
Sesame oil 35	Shandong, China	Aqueous extraction	754.0
Sesame oil 36	Shandong, China	Aqueous extraction	579.3

Sesame oil 37	Shandong,China	Aqueous extraction	620.1
Sesame oil 38	Henan, China	Aqueous extraction	801.8
Sesame oil 39	Chongqing, China	Aqueous extraction	1051.0
Sesame oil 40	Henan, China	Aqueous extraction	892.0
Rapeseed oil 1	Sichuan,China	Hot Pressed	297.7
Rapeseed oil 2	Sichuan,China	Hot Pressed	593.7
Rapeseed oil 3	Sichuan,China	Hot Pressed	429.0
Rapeseed oil 4	Sichuan,China	Hot Pressed	229.0
Rapeseed oil 5	Jiangsu, China	Hot Pressed	193.3
Rapeseed oil 6	Jiangsu, China	Hot Pressed	492.0
Rapeseed oil 7	Shanxi, China	Hot Pressed	54.3
Rapeseed oil 8	Sichuan,China	Hot Pressed	577.0
Rapeseed oil 9	Chongqing, China	Hot Pressed	157.7
Rapeseed oil 10	Jiangsu, China	Hot Pressed	403.2
Rapeseed oil 11	Hubei,China	Hot Pressed	133.5
Rapeseed oil 12	Tianjin,China	Hot Pressed	113.4
Rapeseed oil 13	Neimeng,China	Hot Pressed	-[a]
Rapeseed oil 14	Shanxi,China	Hot Pressed	65.8
Rapeseed oil 15	Fujian, China	Hot Pressed	89.3
Rapeseed oil 16	Chongqing, China	Hot Pressed	118.4
Rapeseed oil 17	Chongqing,China	Hot Pressed	140.0
Rapeseed oil 18	Shanghai, China	Hot Pressed	-
Rapeseed oil 19	Chongqing, China	Hot Pressed	583.3
Rapeseed oil 20	Sichuan, China	Hot Pressed	570.0
Peanut oil 1	Shandong, China	Hot Pressed	94.3
Peanut oil 2	Shandong, China	Hot Pressed	104.2
Peanut oil 3	Shandong, China	Hot Pressed	118.3
Peanut oil 4	Shandong, China	Hot Pressed	82.0
Peanut oil 5	Shandong, China	Hot Pressed	92.3
Peanut oil 6	Shandong, China	Hot Pressed	99.3
Peanut oil 7	Shandong, China	Hot Pressed	181.8
Peanut oil 8	Shandong, China	Hot Pressed	217.0
Peanut oil 9	Shandong, China	Hot Pressed	102.6
Peanut oil 10	Shandong, China	Hot Pressed	118.7
Peanut oil 11	Henan, China	Hot Pressed	78.0
Peanut oil 12	Shandong, China	Hot Pressed	62.9
Peanut oil 13	Shandong, China	Hot Pressed	95.0
Peanut oil 14	Shandong, China	Hot Pressed	85.1

Peanut oil 15	Shandong, China	Hot Pressed	242.3
Peanut oil 16	Shandong, China	Hot Pressed	132.7
Peanut oil 17	Shandong, China	Hot Pressed	90.0
Peanut oil 18	Shandong, China	Hot Pressed	93.0
Peanut oil 19	Shandong, China	Hot Pressed	93.0
Peanut oil 20	Shandong, China	Hot Pressed	117.3

[a] "–" represents below the lowest quantitation level

Table S2. PB experimental design and summary of results.

Method	Extraction temperature (℃) A	Extraction time (min) B	Sample weight (g) C	Desorption temperature (℃) D	Desorption time (min) E	Equilibration time (min) F	Average	SD	RSD
**1**	90	20	7	260	7	10	15881.50	273.65	1.72
**2**	50	50	7	260	3	10	2125.00	152.74	7.19
**3**	50	20	3	260	3	25	607.00	114.55	18.87
**4**	50	50	3	260	7	10	1151.50	53.03	4.61
**5**	90	50	7	240	3	10	36868.00	1173.80	3.18
**6**	50	50	7	240	7	25	1385.00	172.53	12.46
**7**	90	50	3	260	7	25	34040.00	480.83	1.41
**8**	90	50	3	240	3	25	31781.50	2553.36	8.03
**9**	50	20	3	240	3	10	451.50	166.17	36.80
**10**	50	20	7	240	7	25	315.50	53.03	16.81
**11**	90	20	3	240	7	10	12267.00	360.62	2.94
**12**	90	20	7	260	3	25	16300.50	44.55	0.27

Table S3. CCD experimental design and summary of results.

Method	Extraction temperature (℃) A	Extraction time (min) B	Sample weight (g) C	Desorption temperature (℃) D	Desorption time (min) E	Equilibration time (min) F	Average	SD	RSD
**1**	95.86	55	4	280	1.5	15	76539.50	832.26	1.09
**2**	100	60	4	280	1.5	15	93634.00	2153.85	2.30
**3**	100	50	4	280	1.5	15	83357.00	2008.18	2.41
**4**	110	55	4	280	1.5	15	98792.50	2532.15	2.56
**5**	110	55	4	280	1.5	15	100595.50	290.62	0.29
**6**	110	55	4	280	1.5	15	98550.00	4000.81	4.06
**7**	110	48	4	280	1.5	15	99516.00	516.19	0.52
**8**	110	55	4	280	1.5	15	99346.50	453.26	0.46
**9**	110	62	4	280	1.5	15	101759.50	2153.14	2.12
**10**	110	55	4	280	1.5	15	99508.50	4305.57	4.33
**11**	120	50	4	280	1.5	15	85666.50	970.86	1.13
**12**	120	60	4	280	1.5	15	83351.50	1041.57	1.25
**13**	124.14	55	4	280	1.5	15	77122.00	4.24	0.01
